# Emergence of carbapenem-resistant *Serratia marcescens* co-harboring *bla*_NDM-1_, *bla*_KPC-2_, and *bla*_SRT-2_ in bloodstream infection

**DOI:** 10.1128/spectrum.00545-25

**Published:** 2025-09-02

**Authors:** Xuan Wang, Fanghua Fan, Shilei Dong, Yapei Zhang

**Affiliations:** 1 Department of Clinical Laboratory, Zhejiang Hospital 584020, Hangzhou, Zhejiang, People's Republic of China; JMI Laboratories, North Liberty, Iowa, USA

**Keywords:** *Serratia marcescens*, carbapenem resistance, *bla*_NDM-1_, *bla*_KPC-2_, horizontal gene transfer

## Abstract

*Serratia marcescens* is an emerging opportunistic pathogen with high genetic diversity. The emergence and prevalence of carbapenem-resistant *S. marcescens* poses a major health threat due to its intrinsic resistance to multiple antibiotics, which severely restricts the selection and treatment of antibiotics for *S. marcescens* infection. This study presents the first documented case in China of a bloodstream infection caused by *Staphylococcus epidermidis* and *S. marcescens* strain (designated S96) co-producing *bla*_NDM-1_, *bla*_KPC-2_, and *bla*_SRT-2_. Strain S96 exhibited resistance to nearly all categories of β-lactam antimicrobials, β-lactam/inhibitor combinations, aminoglycosides, quinolones, and other clinical antibacterial agents, with the exception of tigecycline. Our main objective was to characterize the genetic mechanisms underlying its carbapenem resistance and plasmid transfer potential. Whole-genome sequencing revealed *bla*_KPC-2_ on a 44,047 bp “IncX6-like” plasmid and *bla*_NDM-1_ on a 100,081 bp IncFII(Yp)-type plasmid, alongside chromosomal *bla*_SRT-2_ and *aac*(6′)−*Ic*. “IncX6-like” and IncFII(Yp)-type plasmids are widely distributed among carbapenem-resistant Enterobacteriaceae strains globally. Conjugation experiments demonstrated that the *bla*_NDM-1_-carrying plasmid could be successfully transferred to recipient *Escherichia coli 600*, with no significant fitness cost observed (*P* > 0.05). The experimental results demonstrate that carbapenem-resistant genes can disseminate among Enterobacteriaceae via plasmid-mediated horizontal transfer between bacterial cells. Comparative genomic analysis revealed plasmid structural homology with global counterparts, demonstrating IS-mediated recombination and horizontal gene transfer. The low adaptive cost of plasmid carriage and multidrug resistance phenotype pose significant challenges for clinical management. This study highlights the need for enhanced clinical surveillance and antibiotic stewardship to curb the spread of such multidrug-resistant pathogens.

**IMPORTANCE:**

Carbapenem resistance in *Serratia marcescens* is primarily mediated by *Klebsiella pneumoniae* carbapenemase (KPC), with New Delhi metallo-β-lactamase (NDM) being a relatively uncommon alternative resistance mechanism. KPC-2 and NDM-1 coexisting in *S. marcescens* is extremely rare clinically. This study reports the first clinical isolate of *S. marcescens* in China co-harboring *bla*_NDM-1_, *bla*_KPC-2_, and *bla*_SRT-2_. The isolate exhibits multidrug resistance to nearly all β-lactam antibiotics and β-lactam/inhibitor combinations, with low adaptive costs and high dissemination potential. The potential spread of resistance genes through mobile genetic elements poses a serious public health risk. The study underscores the need for enhanced surveillance, rational antibiotic use, and novel strategies to combat resistance. It also provides insights into the evolutionary mechanisms of bacterial resistance, emphasizing the urgent need for interventions to address the growing threat of antimicrobial resistance.

## INTRODUCTION

*Serratia marcescens* is recognized to be an important nosocomial pathogen and is usually associated with outbreaks in immunocompromised patients, as well as infants and newborns (1, 2). The infection caused by *S. marcescens* can cause nosocomial infection, affecting several parts of the body, such as the meninges, blood, and lungs, leading to a series of infections like central nervous system infections, blood infections (including endocarditis), and nosocomial pneumonia (3, 4). The European Center for Disease Prevention and Control reported *Serratia* spp. was the sixth most frequent bacterial pathogen causing nosocomial pneumonia and the ninth pathogen isolated from the bloodstream and urinary tract infections, respectively. In recent years, the emergence and spread of acquired antimicrobial resistance (AMR) in *S. marcescens* have become a major health risk to the general public (5). The production of carbapenemases is the primary cause for the rapid and widespread proliferation of *S. marcescens* drug resistance. If infected with carbapenem-resistant *S. marcescens* (CRSM), some patients often have a poor prognosis because *S. marcescens* is intrinsically resistant to ampicillin, nitrofurantoin, tetracycline, macrolides, cefuroxime, cephamycin, and colistin, and there are few antibiotics available.

Similar to other Enterobacteriaceae bacteria, the carbapenemases reported to exist in *S. marcescens* include Class A, Class B, and Class D enzymes. Carbapenem resistance in *S. marcescens* is primarily mediated by *Klebsiella pneumoniae* carbapenemase (KPC), with New Delhi metallo-β-lactamase (NDM) being a relatively uncommon alternative resistance mechanism (6–8). KPC is a member of the class A enzymes and was initially identified in 2001 (9). It has since become one of the most widespread carbapenemases globally, with numerous clinical reports documenting outbreaks among hospitalized patients due to infections caused by KPC-2-producing *S. marcescens* (10, 11). The metallo-β-lactamases (MBLs) belong to Class B enzymes and known as NDM, initially reported in New Delhi, India, and are capable of hydrolyzing nearly all β-lactams. The enzymatic activities of MBLs remain uninhibited by clinically available β-lactamase inhibitors, such as avibactam, relebactam, and vaborbactam. They are more commonly found in carbapenem-resistant *Escherichia coli* and are less frequently detected in *S. marcescens* (12). KPC-2 and NDM-1 coexisting in *S. marcescens* is extremely rare clinically (8, 13), which can broaden the antibiotic resistance spectrum of *S. marcescens*, displaying resistance to nearly all categories of β-lactam antimicrobials, β-lactam/inhibitor combinations, including ceftazidime-avibactam, an important treatment option for infections caused by KPC-producing isolates. This poses a significant threat to the selection and use of antibiotics for patients infected with *S. marcescens*. Here, we first identified a *bla*_KPC-2_ and *bla*_NDM-1_ copositive *S. marcescens* strain in human bloodstream infection (BSI) that was isolated in January 2023 in China, and the complete genetic characteristics were further investigated. This study provides a comprehensive description of the complete genomic features of resistance plasmids of *S. marcescens. bla*_NDM-1_ was embedded within a 100,081 bp IncFII (Yp)-type plasmid, while *bla*_KPC-2_ was inserted into a 44,047 bp “IncX6-like”-type plasmid. We aimed to clarify the transmission and transfer mechanism of carbapenem resistance genes in *S. marcescens* in hospitals to contribute to the treatment of clinical infection and contain the outbreak of nosocomial infection.

## MATERIALS AND METHODS

### Isolate collection and antimicrobial susceptibility testing

Species identification was performed using MALDI-TOF MS (bioMérieux, France). It was further confirmed by whole-genome sequencing (WGS). We determined the minimum inhibitory concentration (MIC) values of strains against common antibiotics according to the broth dilution method recommended by the Clinical and Laboratory Standards Institute (CLSI) (14). MICs for tigecycline and colistin were interpreted using European Committee on Antimicrobial Susceptibility Testing guidelines, while other antibiotics were interpreted using the CLSI M100 33rd Edition. *E. coli* ATCC 25922 was used as controls for testing antimicrobial susceptibility.

### The detection of carbapenem resistance genes

Phenotypic and genotypic detection of carbapenemases was performed using imipenem-EDTA double disk synergy test and NG-Test Carba 5, respectively. The existence of the carbapenemase genes (KPC, NDM, OXA, IMP, and VIM) was confirmed by PCR-based sequencing.

### Bacterial conjugation assay

The transferability of genetic material between bacteria was assessed using the conjugation assay. The S96 strain was labeled as the donor and the rifampin-resistant *E. coli* 600 (EC600) strain as the recipient. Briefly, *S. marcescens* S96 and the recipient were cultured in fresh Luria Bertani (LB) broth at 37°C for 4 h to reach the logarithmic phase. Then, the donor and recipient were mixed at a ratio of 1:4 and inoculated on an LB agar. After culturing for 24 h at 37°C, the bacterial mixture was incubated on Mueller-Hinton agar (Oxoid, Hampshire, UK) plates containing 200 mg/L rifampin and 2 mg/L meropenem. Antimicrobial susceptibility testing and PCR amplification of the transconjugants were then performed to demonstrate the successful transfer of the plasmid from the donor to the recipient.

### Determination of growth rate

To construct an *in vitro* growth curve, all tested strains were inoculated into 5 mL of LB broth and shaken at 37°C and 250 rpm overnight. Bacterial culture was then adjusted to OD_600_ of 0.1 and 1,000-fold diluted in 5 mL LB, followed by incubating at 37°C with shaking (250 rpm). Viable cells were determined by 10 µL of broth being spread onto Mueller-Hinton medium and incubated 12–18 h. After dilution, the serial dilutions were spread on LB agar at 0, 1, 2, 3, 4, 5, 6, 7, 8, 9, 10, 11, 12, and 24 h. Three independent experiments were performed for each strain. Values of growth curves were analyzed by one-way ANOVA with GraphPad Prism 9. The values returning an adjusted *P* value of 0.05 were considered significant. Growth curves were plotted with GraphPad Prism version 8.3.0 according to the mean values of and incubation time.

### WGS analysis

The bacterial whole genome DNA was extracted by QIAamp DNA Mini Kit (Qiagen, Valencia, CA, USA). Purified DNA samples were submitted to next-generation high-throughput sequencing on the HiSeq2000 platform (Illumina, San Diego, CA, USA). The strain was further subjected to long-read high-throughput sequencing on the MinION platform (Nanopore, Oxford, United Kingdom). Sequence assembly was performed using the Unicycler software and Prokka v.1.13. The Rapid Annotation using Subsystems Technology annotation website server 2.0 (https://rast.nmpdr.org/rast.cgi) was used to annotate the genomes. Plasmid replicons and AMR genes were predicted through the PlasmidFinder (https://bitbucket.org/genomicepidemiology/plasmidfinder/src/master/) and ResFinder website (http://genepi.food.dtu.dk/resfinder), respectively. Sequence comparison of plasmids was conducted using BLAST Ring Image Generator (BRIG) and Easyfig.

## RESULTS

### Isolate collection and antimicrobial susceptibility testing

**Fig 1:**
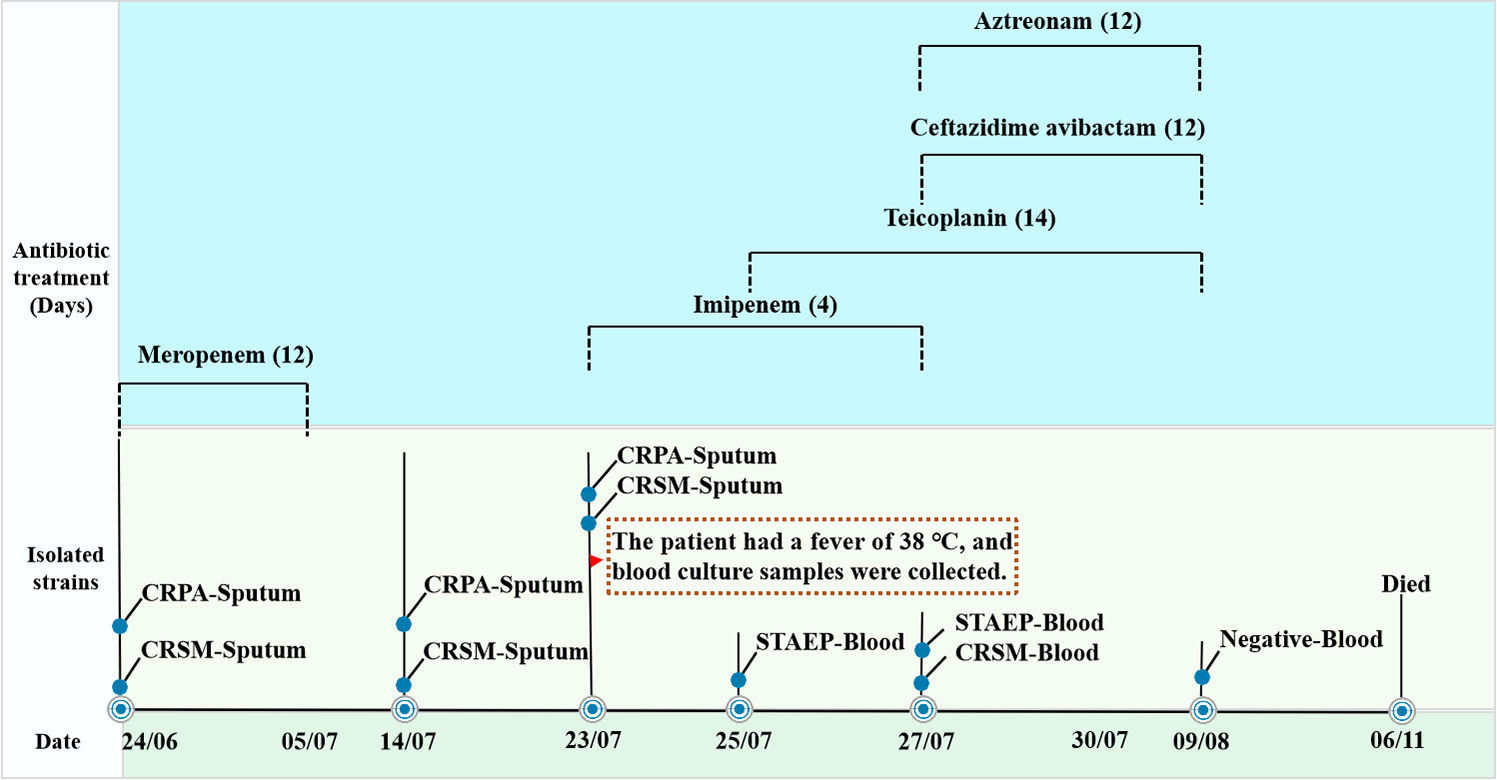
Clinical timeline of the patient. Days of antibiotic treatment are indicated by the blue box on the black line above the timeline. The timeline of this hospitalization is not drawn to scale. CRPA respects carbapenem-resistant *Pseudomonas aeruginosa*. STAEP respects *Staphylococcus epidermidis*.

*S. marcescens* S96 was isolated from a blood sample of a hospitalized male patient in Hangzhou, Zhejiang Province, China, in 2023. The patient was a 96-year-old male who was sent to the intensive care unit for acute pancreatitis. The patient was accompanied by many underlying diseases such as Type II respiratory failure, renal insufficiency, and hypertension. Prior to this hospitalization, he had been admitted to the ICU of this hospital multiple times and received multiple anti-infective treatments including imipenem, cefoperazone/sulbactam, and polymyxin B. During the current admission, sputum cultures revealed colonization with CRPA and CRSM. Given the patient’s compromised status, the clinical team initiated a 12-day prophylactic course of meropenem (Fig. 1). On hospital day 37, the patient had a fever of 38°C, with subsequent blood cultures sequentially isolating STAEP followed by CRSM (Fig. 1). When there is a BSI, the white blood cell count is 12.2 × 10^9^ /L, Serum Amyloid A **>** 320 mg/L, C-reactive protein 95.96 mg/L. The treating physicians initiated empirical antimicrobial therapy with imipenem to control the infection. Based on the subsequent drug susceptibility testing results of STAEP and CRSM, the patient was initiated on combination therapy with teicoplanin, ceftazidime-avibactam, and aztreonam, which initially achieved infection control (Fig. 1). However, the clinical course was complicated by recurrent BSIs despite continued antimicrobial therapy. The patient’s condition progressively deteriorated, culminating in multiple organ failure and eventual mortality.

The antimicrobial susceptibility profiles of *S. marcescens* S96 and transconjugants EC600: pS96-NDM-1 are presented in Table 1. S96 was intermediate to tigecycline, but resistant to imipenem, meropenem, amikacin, levofloxacin, cefoperazone–sulbactam, ceftazidime-avibactam, and Polymyxin B.

**TABLE 1:** Drug sensitivity results of the strains involved in this study^*a*^

Strain ID	IMP	MEM	AMK	LEV	SCF	CZA	PB	TCG
S96	≥256	≥32	≥64	2	≥64	≥256	≥256	4
EC600:pS96-NDM-1	8	8	≥64	0.5	≥64	≥256	0.125	≤0.5
EC600	≤0.25	≤0.25	≤2	0.5	≤8	0.125	≤0.064	≤0.5
ATCC25922	≤0.25	≤0.25	≤2	≤0.125	≤8	0.125	≤0.064	≤0.5

^*a*^ AMK, amikacin; CZA, ceftazidime-avibactam; IPM, imipenem; LEV, levofloxacin; MEM, meropenem; MIC, minimum inhibitory concentration; PB, Polymyxin B; SCF, cefoperazone-sulbactam; TGC, tigecycline.

### Carbapenemase-encoding genes and conjugation experiments

PCR-based sequencing demonstrated the presence of *bla*_NDM-1_ and *bla*_KPC-2_ in *S. marcescens* strain S96. The *bla*_NDM-1_-carrying plasmid was successfully transferred from *S. marcescens* strain S96 to EC600, making the conjugants resistant to imipenem and meropenem. Compared to the recipient EC600, the meropenem and imipenem MICs of conjugants increased at least 32-fold, respectively (Table 1). However, the *bla*_KPC-2_-harboring plasmid failed to transfer to the recipient strain in our study.

### Fitness cost change of transconjugants

**Fig 2:**
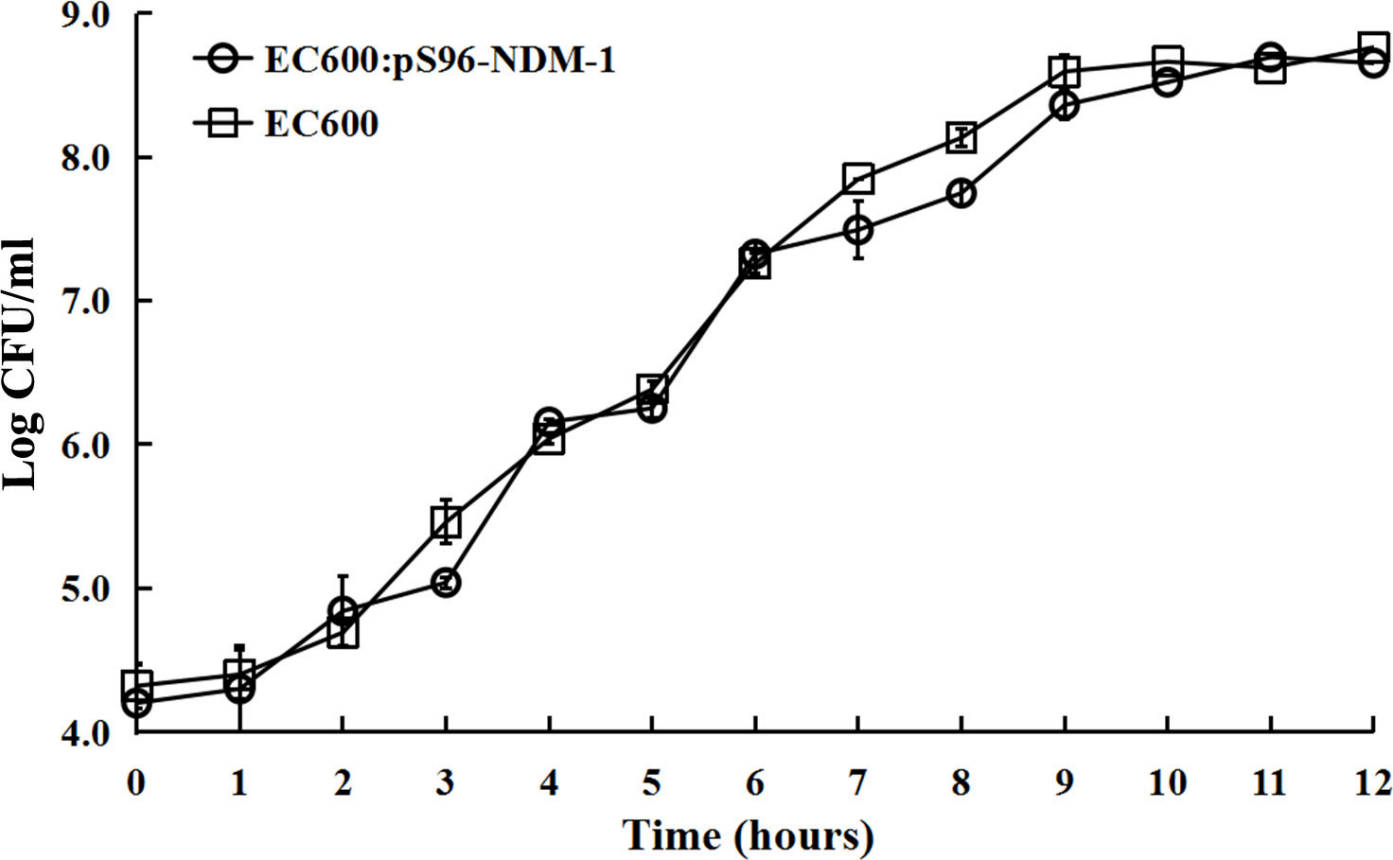
Growth curves of EC600:pS96-NDM-1 and Ec600. No significant differences in bacterial counts were detected across the examined time points.

To further investigate whether these highly stable plasmids have an effect on the fitness cost of strains, we explored the growth curve of EC600 and its transconjugant EC600: pS96-NDM-1. No significant growth rate difference was observed (*P* > 0.05, Fig. 2), indicating that NDM-1 plasmid acquisition incurred minimal fitness cost.

### Genomic characterization of the bacterial chromosomes

We submitted the complete nucleotide sequences of the genomes we sequenced to GenBank (accession number JBMGQM010000000). The genome size of strain S96 was 5,749,368 bp, with a G + C content of 59.45% and comprizing 5319 coding sequences (CDS) and 85 tRNAs and 5 CRISPR-Cas. The genome of S96 also included five plasmids: an IncFII(Yp)-type plasmid pS96-NDM-1, an “IncX6-like”-type plasmid pS96-KPC-2 and 75,066 bp plasmids, as well as two small plasmids (<10 kb) (Table 2). KEGG pathway analysis found that S96 contains 76 Drug resistance genes (Fig. S1). The resistance comprised various AMR genes conferring resistance to carbapenems, cephalosporins, aminoglycosides, sulfonamides, chloramphenicol, and fluoroquinolones.

**TABLE 2:** Molecular characterization of genome from S96 strain

Location	Plasmid type	Sequence length (bp)	GC content (%)	Accession number	Accession strain name	Accession plasmid name	Identity (%)
S96-KPC	IncX6-like	44,047	49.84	MN904741.1	*S. marcescens*	pHENAN1602-KPC	100
S96-B	–^*a*^	75,066	53.85	CP028178.1	*K. pneumoniae*	pGMI16-006_2	99.92
S96-NDM	IncFII (Yp)	100,081	55.58	MF042351.1	*S. marcescens*	pNDM_12TM	99.92
S96-G	IncFII (S)	2,118	53.59	CP053379.1	*S. marcescens*	p1	92.34
S96-F	Col440I	4,096	55.52	CP044113.1	*K. michiganensis*	unnamed4	100

^*a*^ –: No matching plasmid replicon types identified in NCBI database.

### Characterization of the pS96-NDM and pS96-KPC plasmids

**Fig 3:**
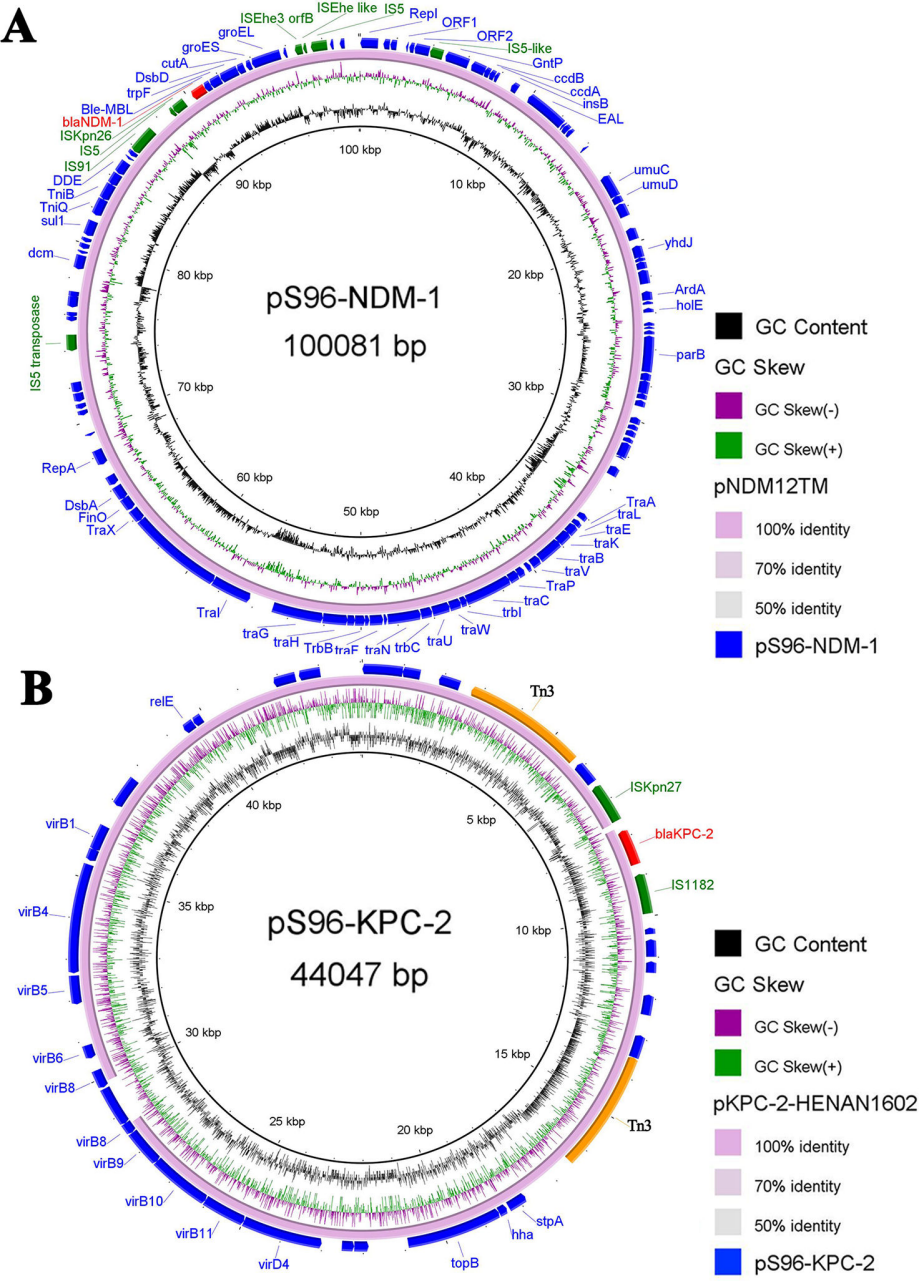
The circular maps of pS96-NDM-1 and pS96-KPC-2. (**A**) The circle in pink represented plasmid pNDM12TM. (**B**) The circle in pink represented pKPC-2-HENAN1602. The peak map in (**A and B**) represented the GC content of plasmid pS96-NDM-1 and pS96-KPC-2, respectively. Arcs in red indicate the position of *bla*_NDM_ and *bla*_KPC_ in plasmids pS96-NDM-1 and pS96-KPC-2. The maps were created by BRIG v0.95.

**Fig 4:**
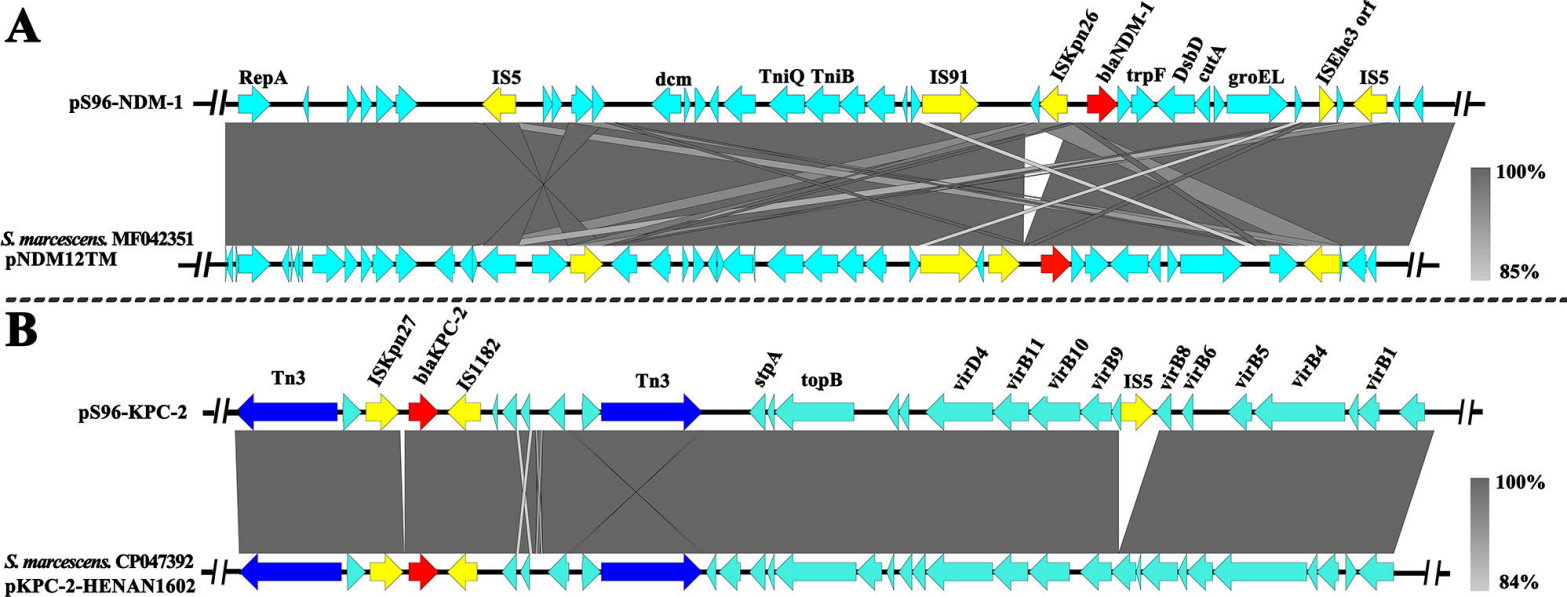
Comparative analysis of the *bla*_NDM-1_ and *bla*_KPC-2_-harboring plasmids. (**A**) Sequence alignment analysis among plasmids pS96-NDM-1 and pNDM12TM (GenBank accession no. MF042351), (**B**) Sequence alignment analysis among plasmids pS96-KPC-2 and pKPC-2-HENAN1602 (GenBank accession no. CP047392). Red represents the antibiotic resistance genes. Yellow and blue represent the insertion sequences and transposons, respectively.

According to the sequencing results of pS96-NDM-1, it was a 100,081 bp plasmid belonging to the IncFII (Yp)-type, with GC content of 55.58% (Fig. 3A). This targeted plasmid contained 110 open reading frames (ORFs). Two resistance genes *bla*_NDM-1_ and *sul1* were identified in pS96-NDM-1, conferring resistance to carbapenems and sulfonamides, respectively. Blast comparison indicates that pS96-NDM-1 in this study shares extensive similarity with pNDM12TM (99% nucleotide identity and query coverage), an IncFII (Yp)-type plasmid with the length of 119,046 bp in a CRSM strain isolated from a Romanian hospital in 2015 (15). Like the source of our strain, pS96-NDM-1 and pNDM12TM both possess the conserved IncFII (Yp)-type backbone regions. There are two major genetic differences between the backbones of pS96 NDM-1 and pNDM12TM (Fig. 4A). First, compared to the plasmid pNDM12TM, a new insertion sequence IS*Kpn26* has been added in front of the *bla*_NDM-1_ gene. Second, in pS96-NDM-1, a structural region of about ~19 kb is truncated, including multiple hypothetical proteins, peptidases, and integrases. Moreover, 6 bp TSDs ([target site duplications], TCTAGA) were observed upstream of *bla*_NDM-1_ genetic elements. The other 8 bp TSDs (CCTCGAGG) were identified downstream of the IS *26* genetic elements in pS96-NDM-1.

According to the sequencing results of pS96-KPC-2, it was a 44,047 bp plasmid belonging to the IncX6-like type, with GC content of 49.84% (Fig. 3B). This plasmid contained 36 ORFs. Among the 36 ORFs, only one antibiotic resistance gene *bla*_KPC-2_ was found after comparison with the Antibiotic Resistance Genes Database. The *bla*_KPC-2_ gene was flanked by an IS1182 element downstream and an IS*Kpn27* gene upstream. Comparative plasmid analysis revealed that pS96-KPC-2 shares a high identity with pKPC-2-HENAN1602, a IncX6-like type plasmid with the length of 42,724 bp (16). Compared to pKPC-2-HENAN1602, the IS5 family transposase is inserted between VirB8 and VirB9 in pS96-KPC-2 (Fig. 4B).

## DISCUSSION

*S. marcescens* is an important health-related pathogen, and in recent years, reports of CRSM have been increasing, with many countries or regions experiencing epidemic spread (6, 10, 11, 15). Here, we describe the microbiological and clinical characteristics of BSIs caused by CRSM S96. This strain not only contains carbapenem-resistant genes *bla*_KPC-2_ and *bla*_NDM-1_, but also carries β-lactamase resistance gene *bla*_SRT-2_ and aminoglycoside resistance gene *aac (2)-Ic*, owing to its simultaneous production of *bla*_KPC-2_, *bla*_NDM-1_, *bla*_SRT_-2, and *aac (2)-Ic. S. marcescens* S96 exhibits a highly drug-resistant phenotype, showing resistance to nearly all categories of β-lactam antimicrobials, β-lactam/inhibitor combinations, aminoglycosides, quinolones, and other clinical antibacterial agents with the exception of tigecycline, thereby significantly elevating the complexity of treatment for infected patients.

KPC-2 and NDM-1 are predominantly disseminated among bacterial populations through plasmids. Specifically, KPC-2 primarily exists on plasmids ranging from 50 to 118 kb in size, belonging to various incompatibility groups, including IncF, IncN, IncR, IncK, IncL/M, and IncX, most of which are conjugative plasmids (17–21). The KPC-2 gene carried by the strain isolated in this study is present in a plasmid that cannot be classified, which is named a “IncX6-like” plasmid, and this plasmid named pCP40 was defined as the first report of this “untypeable” plasmid isolated from *Citrobacter portucalensis* (22). Subsequently, the plasmid was found in *E. coli* and *Citrobacter freundii* (23). In 2020, for the first time, an “IncX6-like” plasmid was reported in *S. marcescens* from the Henan region of China, and it was named pKPC-2HENAN1602 (16), pS96-KPC-2 and pKPC-2HENAN1602 have almost the same antibiotic resistance gene region and different backbone sequences of plasmids, but the ORFs encoding the key functions of plasmid replication, conjugation, transport, and stability and content of the backbone of the plasmid certainly resemble each other. These results showed that pS96-KPC-2 and pKPC-2HENAN1602 have a certain evolutionary relationship, which proved that the plasmid has a strong recombination and horizontal transfer ability. The plasmid isolated from the strains in this study shares a similar plasmid backbone with the IncFII(Yp)-type plasmid pNDM12TM. For NDM-1, the main types of plasmids carrying *bla*_NDM-1_ include IncHI2, IncFII, IncFIA, and IncX3 (21, 24, 25), IncFII(Yp)-type plasmid was first isolated from a *Klebsiella oxytoca* strain in Taiwan. Subsequently, reports of the plasmid carrying the *bla*_NDM-1_ gene also emerged from Vietnam and South Africa (26–28). While both types of plasmids have been identified in *S. marcescens*, there is a dearth of reports, both domestically and internationally, documenting their coexistence within a single strain without incurring adaptive costs. To our knowledge, no studies have documented the simultaneous presence of “IncX6-like” plasmids and IncFII (Yp)-type plasmids in *S. marcescens* on a global scale. The origin of this particular strain remains obscure. Nonetheless, our research posits that this phenomenon could be linked to the patient’s extended stay in the ICU and the administration of multiple antibiotic regimens (Fig. 1), echoing the scenario detailed by Zhang et al. (13).

Plasmids impose an additional metabolic burden on the host, which typically results in adaptive costs that are detrimental to bacterial competition and plasmid dissemination (29). However, some studies have also revealed that the adaptive cost to the host of plasmids carrying carbapenem-resistance genes in Enterobacteriaceae bacteria is relatively low (30, 31). In our study, the growth profiles of the recipient strain EC600 and the conjugant EC600: pS96-NDM-1 were found to be statistically indistinguishable, suggesting that strains harboring the pS96-NDM-1 plasmid incur minimal adaptive costs. This experiment result is generally consistent with previous research by Huang et al. (30), which indicates that even when *K. pneumoniae* produce KPC-2 and also carry NDM-5, the adaptive cost remains negligible. Further exploration is warranted to elucidate the mechanisms behind this observation. The combination of multidrug resistance and a low adaptive cost confers a substantial survival advantage to the strain in its environmental niche.

Genetic environment analysis revealed that the *bla*_KPC-2_ gene was located in a 44,047 bp plasmid belonging to the IncX6-like type, which was located in the core region of antibiotic resistance and was composed of Tn3 family transposons, recombinant enzyme genes, IS*Kpn6,* and IS*Kpn27*. The region including *bla*_KPC-2_ formed a ‘Tn3-IS*Kpn6-bla*_KPC_-IS*Kpn27*-Tn3’ structure called the core *bla*_KPC_ platform (16), which was an independent region as a movable element and belongs to transposon Tn6296 and its derivatives. Despite the unsuccessful acquisition of transconjugants containing the pS96-KPC-2 plasmid in our experimental conditions, the potential for horizontal gene transfer cannot be definitively excluded. Mobile genetic elements (MGE), including ISs, integrons, and transposons, play a particularly important role in the resistance gene transfer among different species (32), participating in several phenomena that involve gene acquisition (33). Tn3 mobile genetic element was consistently found in many other plasmids, which have the ability to mobilize specific genomic DNA as part of the composite transposons (34). Our recent study also provided direct evidence of plasmid fusion via ISs between two different bacterial species within one patient (35).

*bla*_NDM-1_ were located in 100,081 bp plasmid belonging to the IncFII(Yp)-type. Similar to *E. coli* and *K. pneumoniae* among other Enterobacteriaceae, the strains identified in this study were found to contain the 'IS*Aba125-bla*_NDM_-*bla*_MBL_-trpF-dsbD' domain. The presence of this domain in *S. marcescens* suggests that the *bla*_NDM-1_ gene may share a common origin with other Enterobacteriaceae bacteria. Conjugation experiments investigated the transferability of pS96-NDM-1. Taken together, the transfer of resistance gene *bla*_NDM-1_ could occur through MGE and/or conjugation. Moreover, an analysis of 236 global carbapenemase-producing *S. marcescens* (CPSM) genomes, retrieved from NCBI database, revealed that *bla*_KPC-2_-harboring *S. marcescens* maintained a long-term spread and *S. marcescens* easily acquired carbapenemase genes by plasmid transfer (13). It is worth noting that the patient was treated with long-term multiple antibiotics. We speculate that under antibiotic selection pressure, *bla*_KPC-2_-harboring *S. marcescens* forms the concurrence of two carbapenemase genes by obtaining the pS96-NDM-1. Even more worryingly, clonal dissemination of CPSM, indicating the vertical spread of carbapenemase genes, is occasionally reported in human, food-producing animals, environment, and so on (6, 13, 36, 37), which also plays an important role in the evolutionary history of carbapenem resistance in *S. marcescens*.

With regard to *bla*_SRT-2_, an AmpC-type β-lactamase gene located on a chromosome confers resistance to cephalosporin, which was first reported in a *S. marcescens* strain in 2004 (38) and demonstrates a frequent co-occurrence with other resistance determinants in *S. marcescens*, such as *aac (6′)-Ic*, *bla*_CTX-M-3_, and *bla*_KPC-2_ (39), a pattern that is consistent with our current findings. *aac (6′)-Ic* is an aminoglycoside resistance gene, but unlike 16S rRNA methylases, which confer high-level resistance to most aminoglycosides (40). One important point is that although these mechanisms cause only low-level resistance, they usually constitute the first step in the development of high-level resistance, because bacteria carrying these genes have an adaptive advantage compared to the highly susceptible bacterial population in environments with low concentrations of this antimicrobial group (41). Furthermore, exposure to new classes of antibiotics not only drives the selection of specific resistance mechanisms but also facilitates the development of other resistance mechanisms, the phenomenon well-documented in a classical review on the evolution of AMR (42).

In summary, to the best of our knowledge, this study represents the first documented case in China of BSI caused by *S. marcescens* co-harboring two carbapenem resistance genes located on plasmids, along with chromosomally encoded aminoglycoside and cephalosporin resistance genes. Our findings demonstrate the acquisition of carbapenem resistance determinants and reveal the potential dissemination patterns of multidrug-resistant phenotypes with complex genetic backgrounds. The convergence of multiple carbapenemase genes, coupled with their continuous evolution and dissemination, may exacerbate the development of carbapenem resistance and contribute to the escalation of multidrug resistance in *S. marcescens* populations. Consequently, there is a critical need for enhanced clinical surveillance of such strains and resistance determinants, particularly to curb the spread of nosocomial infections.

## SUPPLEMENTAL MATERIAL

Fig. S1 spectrum.00545-25-s0001.docx KEGG-based distribution of protein-coding sequences in each subsystem.

ASM does not own the copyrights to Supplemental Material that may be linked to, or accessed through, an article. The authors have granted ASM a non-exclusive, world-wide license to publish the Supplemental Material files. Please contact the corresponding author directly for reuse.
