# Supplementary material for: Emergence of carbapenem-resistant Serratia marcescens co-harboring blaNDM-1, blaKPC-2, and blaSRT-2 in bloodstream infection
Source: Microbiol Spectr. 2025 Sep 2;13(10):e00545-25. doi: 10.1128/spectrum.00545-25 (PMC12502704; doi:10.1128/spectrum.00545-25)
Supplement: Fig. S1 — KEGG-based distribution of protein-coding sequences in each subsystem. [file spectrum.00545-25-s0001.docx]

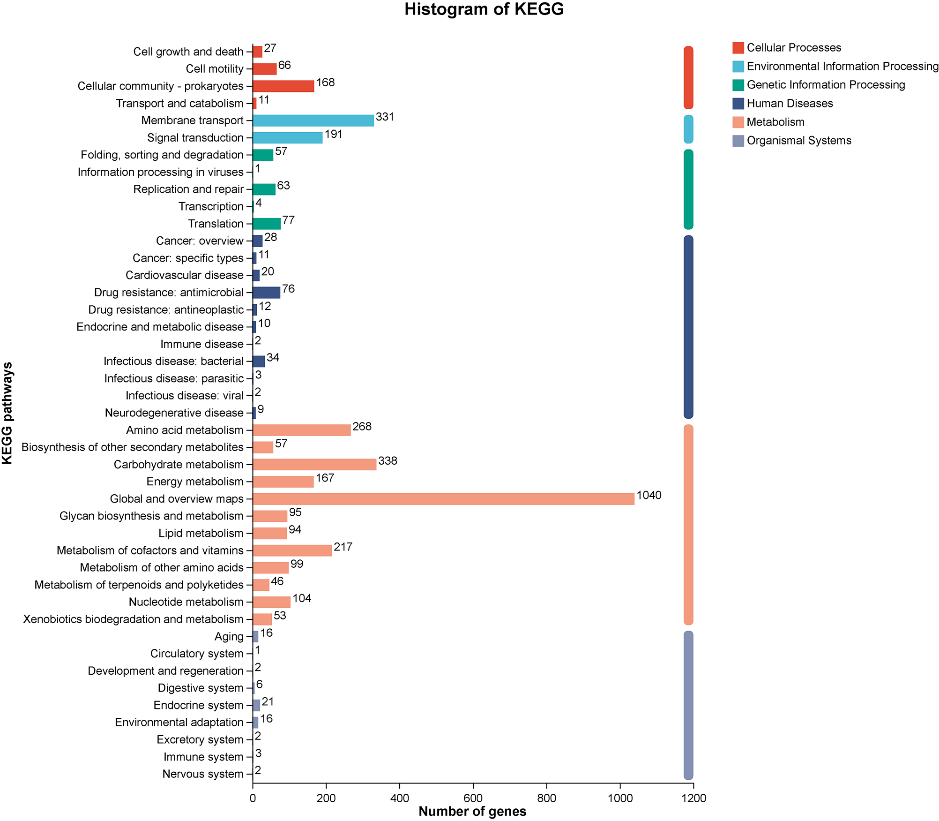


**Fig S1** KEGG-based distribution of protein-coding sequences in each subsystem. The number of sequences in each subsystem category is shown on the right of each column. Among the genes associated with human diseases, drug resistance genes were the most prevalent, accounting for a total of 76 identified genes.
